# Development of a Novel Electrochemiluminescence ELISA for Quantification of α-Synuclein Phosphorylated at Ser^129^ in Biological Samples

**DOI:** 10.1021/acschemneuro.2c00676

**Published:** 2023-03-15

**Authors:** Suman Dutta, Simon Hornung, Hash Brown Taha, Karl Biggs, Ibrar Siddique, Lea M. Chamoun, Hedieh Shahpasand-Kroner, Carter Lantz, Marcos Herrera-Vaquero, Nadia Stefanova, Joseph A. Loo, Gal Bitan

**Affiliations:** ^†^Department of Neurology, David Geffen School of Medicine, ^‡^Department of Integrative Biology & Physiology, and ^§^Department of Chemistry and Biochemistry, University of California, Los Angeles, California 90095, United States; ∥ Division of Neurobiology, Department of Neurology, Medical University of Innsbruck, 6020 Innsbruck, Austria; ^⊥^Brain Research Institute and ^#^Molecular Biology Institute, University of California, Los Angeles, California 90095, United States

**Keywords:** Electrochemiluminescence ELISA, α-synuclein, phosphorylation, pS129, biomarker

## Abstract

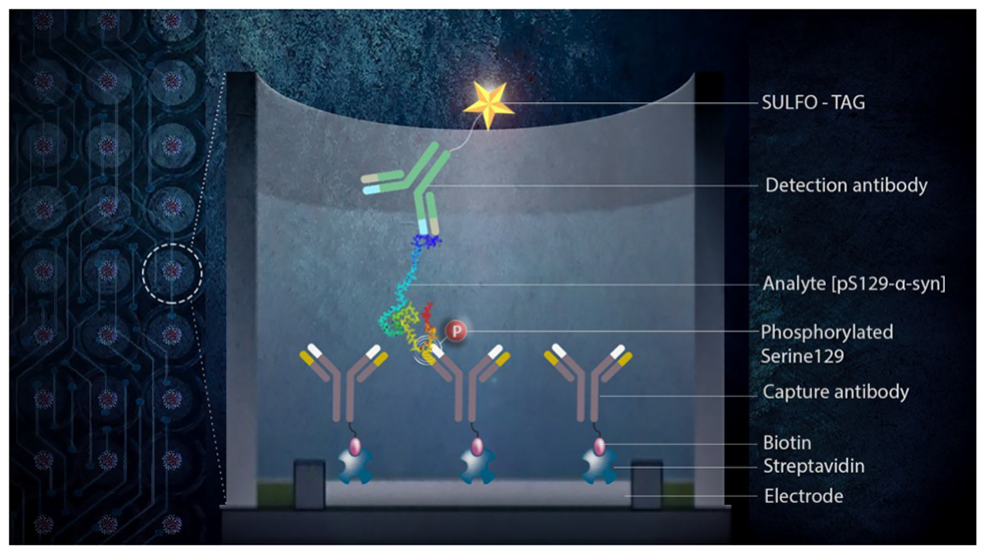

Synucleinopathies are a group of neurodegenerative diseases including Parkinson’s disease (PD), dementia with Lewy bodies (DLB), and multiple system atrophy (MSA). These diseases are characterized by the aggregation and deposition of α-synuclein (α-syn) in Lewy bodies (LBs) in PD and DLB or as glial cytoplasmic inclusions in MSA. In healthy brains, only ∼4% of α-syn is phosphorylated at Ser^129^ (pS^129^-α-syn), whereas >90% pS^129^-α-syn may be found in LBs, suggesting that pS^129^-α-syn could be a useful biomarker for synucleinopathies. However, a widely available, robust, sensitive, and reproducible method for measuring pS^129^-α-syn in biological fluids is currently missing. We used Meso Scale Discovery (MSD)’s electrochemiluminescence platform to create a new assay for sensitive detection of pS^129^-α-syn. We evaluated several combinations of capture and detection antibodies and used semisynthetic pS^129^-α-syn as a standard for the assay at a concentration range from 0.5 to 6.6 × 10^4^ pg/mL. Using the antibody EP1536Y for capture and an anti-human α-syn antibody (MSD) for detection was the best combination in terms of assay sensitivity, specificity, and reproducibility. We tested the utility of the assay for the detection and quantification of pS^129^-α-syn in human cerebrospinal fluid, serum, plasma, saliva, and CNS-originating small extracellular vesicles, as well as in mouse brain lysates. Our data suggest that the assay can become a widely used method for detecting pS^129^-α-syn in biomedical studies including when only a limited volume of sample is available and high sensitivity is required, offering new opportunities for diagnostic biomarkers, monitoring disease progression, and quantifying outcome measures in clinical trials.

## Introduction

Synucleinopathies, including Parkinson’s disease (PD), dementia with Lewy bodies (DLB), and multiple system atrophy (MSA), are characterized clinically by a chronic and progressive decline in motor, cognitive, behavioral, and/or autonomic functions. Deposits of fibrillar α-synuclein (α-syn) as Lewy bodies (LBs) and Lewy neurites in PD and DLB or as glial cytoplasmic inclusions (GCIs) in MSA are pathological hallmarks of these diseases.^1,2^ Differential diagnosis of synucleinopathies is difficult due to clinical symptoms overlap, especially in early disease stages.^3^ Phosphorylated forms of α-syn, particularly at Ser129 (pS^129^-α-syn) are highly enriched in LBs and GCIs and are thought to be related to the disease process.^4,5^ Therefore, if pS^129^-α-syn can be measured in bodily fluids of patients, it could serve as a sensitive biomarker for improving diagnosis accuracy, measuring disease progression, and assessing therapeutic outcomes.^6,7^

Currently, commercially available assays for quantifying pS^129^-α-syn are limited. Cell-based ELISAs are available from Abexxa (Cambridge, United Kingdom), Creative Diagnostics (Shirley, NY), and Aviva Systems Biology (San Diego, CA), yet based on the information provided by the companies, these assays are not optimized for detecting the recombinant protein in human samples available in limited quantities and suffer from intrinsically high variability, mainly due to the difficulty of plating the exact number of cells in each well and to variation in cell proliferation rate and responsivity. A typical sandwich ELISA is sold by MyBioSource,^8^ yet the company’s Web site disclaims “cross-reaction to other targets may potentially exist” and “cross-reactivity could vary between sample type or species”. In addition, considering the low concentrations of pS^129^-α-syn in human biofluid samples, as reported in earlier studies,^9,10^ the sensitivity limit of the assay, 3.12 ng/mL, likely would not allow reliable measurement in human biofluids.

Previously, several groups have reported assays for pS^129^-α-syn, which varied widely in sensitivity, and used them to measure the phospho-protein in human cerebrospinal fluid (CSF),^10−12^ plasma,^12−14^ human cell culture lysates,^13^ human and rat brain lysates,^13^ and human erythrocytes.^15^ The assays included traditional ELISA^6,11,14^ and higher-sensitivity methods, such as Singulex Erenna,^12^ Luminex,^10^ and AlphaLISA.^13^ Recently, the Zhang group created a pS^129^-α-syn electrochemiluminescence ELISA (ECLIA) using a biotinylated anti-pS^129^-α-syn antibody (BioLegend, San Diego, CA) and anti-α-syn antibody clone 42 (BD Bioscience, CA) labeled with Sulfo-TAG. Applying this assay, they quantified the concentration of pS^129^-α-syn in the membrane and cytosolic fractions of erythrocytes isolated from blood samples of healthy controls (HC) and patients with PD.^15^ Despite the importance of pS^129^-α-syn as a potential biomarker for diagnosis and measuring the progression of synucleinopathies, none of these assays have become mainstream.

Three main issues likely have prevented the general use of the aforementioned assays. First, the specificity and reproducibility of the antibodies used varied and some of the antibodies were not readily available to other research groups. Second, in most of the studies published to date, the standard curve was generated using recombinant α-syn phosphorylated *in vitro* by casein kinase II^4,6,10,11^ or polo-like kinase 2,^13^ raising concerns regarding the completion of the phosphorylation at Ser^129^ and possible phosphorylation of other sites, which might cross-react with the antibodies used in the assay or otherwise affect their binding to pS^129^-α-syn.^16^ Third, most studies published to date focused on demonstrating the suitability of their assays only for a specific sample type, e.g., CSF,^10,11^ but did not report testing of different antibody combinations, fine-tuning the protocol, or comparing the assay in different biofluids. To address these concerns, we have extensively researched publications on commercially available antibodies and used as a standard the semisynthetic pS^129^-α-syn first reported by the Lashuel group^17^ and made available by the Michael J. Fox Foundation via Proteos Inc. We have considered multiple factors in selecting the antibodies, tested several methodological approaches, and evaluated the suitability of our assay to quantify pS^129^-α-syn in several commonly analyzed biological sample types. A comparable study was published recently by Cariulo et al. in which they characterized different antibody combinations of anti-α-syn and anti-pS^129^-α-syn antibodies and measured total and pS^129^-α-syn in CSF and plasma using a Singulex Erenna immunoassay.^12^

The novel ECLIA we describe here for measurement of pS^129^-α-syn detects pg/mL concentrations of pS^129^-α-syn and has a wide dynamic linear range and low intra- and interassay variability. It is suitable for use in multiple types of biological samples and thus provides a platform for measuring pS^129^-α-syn as a biomarker for a variety of clinical and research applications.

## Results

### Evaluation of Capture and Detection Antibody Pairs and Cross-Reactivity with Unphosphorylated α-Syn

We chose to use the MSD ECLIA platform for the development of a pS^129^-α-syn assay as it offers high-sensitivity detection at a relatively affordable price and has become widely used in academic institutions, the biotechnology industry, and pharmaceutical companies. The detection principle in this method is based on light emission from electrochemiluminescent labels conjugated to the detection antibody upon application of voltage to the carbon electrodes printed on the back of the wells. The analyte is captured by biotinylated antibodies bound to a streptavidin-coated plate surface. Having made this choice, we tested different combinations of capture and detection antibodies for the degree of sensitivity and reproducibility of the measurements. A number of antibodies with variable sensitivity and specificity have been developed for specific recognition of pS^129^-α-syn, some of which are commercially available.^18^ A recent study comparing several such antibodies found that the rabbit monoclonal antibody EP1536Y had the highest sensitivity and specificity for pS^129^-α-syn,^19^ consistent with a previous study.^20^ Some concerns about cross-reactivity of this antibody have been raised in a bioRxiv manuscript by Arlinghaus et al.^21^ but were not reproduced in a recent, thorough characterization of six anti-pS^129^-α-syn antibodies by Lashuel et al.^18^ Therefore, we decided to use this antibody either for capture or for detection in combination with an antibody that recognizes α-syn regardless of its phosphorylation status. Reports in the literature and our own experience suggested that mAb MJFR1^22^ might be a good candidate for this task. In addition, we tested the anti-human α-syn antibody supplied with the commercial ECLIA kit sold by MSD.

We tested three different combinations of capture and detection antibodies (Table 1). In two combinations, EP1536Y was used either as a biotinylated capture antibody paired with Sulfo-tag anti-human α-syn (MSD) as the detection antibody or as a sulfonated detection antibody combined with a biotinylated MSD anti-human α-syn capture antibody. In the third combination, biotinylated MJFR1 was used for capture, and Sulfo-tag EP1536Y for detection.

**Table 1:** Evaluation of Antibody Combinations for Development of the pS^129^-α-Synuclein Electrochemiluminescence ELISA^a^

capture antibody	detection antibody	LoB (pg/mL)	LLoD (pg/mL)	LLoQ (pg/mL)	ULoQ (pg/mL)	intra-assay CV (%)	interassay CV (%)
biotin-EP1536Y (anti-pS^129^-α-syn)	anti-human α-syn-Sulfo tag (MSD)	3.1 ± 0.6	6 ± 3	14 ± 10	66,167	3.9	7.2
biotin-anti-human α-syn (MSD)	EP1536Y-Sulfo tag	11 ± 3	15 ± 8	75 ± 13	33,088	4.6	10.5
biotin-MJFR1 (anti-human α-syn)	EP1536Y-Sulfo tag	11 ± 10	32 ± 10	97 ± 30	33,088	7.2	28.3

a Mean ± SD are shown for LoB, LLoD, and LLoQ.

**Figure 1:**
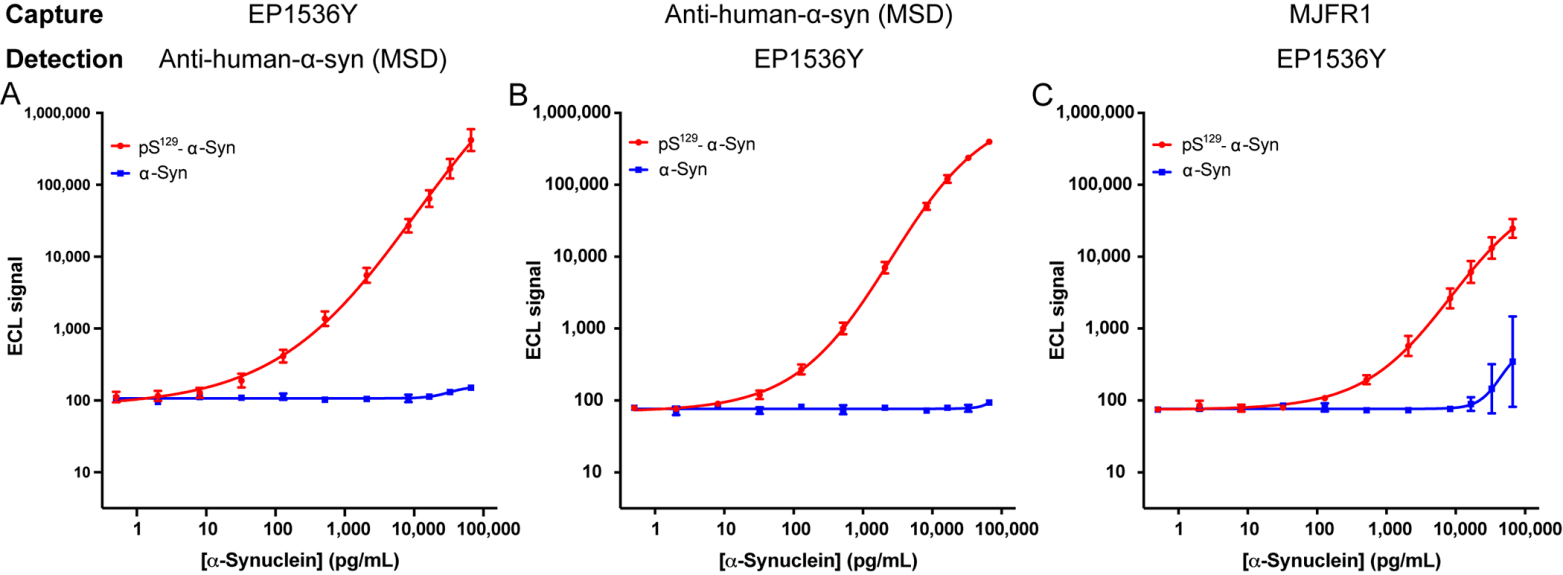
Standard curves for pS^129^-α-syn (red) and unphosphorylated α-syn standards (blue) using different antibody configurations. (A) Biotinylated EP1536Y used for capture and Sulfo-tagged MSD’s anti-human-α-syn antibody for detection. (B) Biotinylated MSD’s anti-human-α-syn antibody used for capture and Sulfo-tagged EP1536Y for detection. (C) Biotinylated mAb MJFR1 was used for capture and Sulfo-tagged EP1536Y for detection. Representative standard curves (mean ± SD of two technical replicates) of at least five independent experiments are shown.

The sensitivity and reproducibility of each antibody combination were evaluated based on a standard curve generated using the semisynthetic pS^129^-α-syn at concentrations ranging from 0.5 to 66 167 pg/mL. Unphosphorylated α-syn standards were used to evaluate the specificity of the assay for the phosphorylated form. Each antibody combination was evaluated in at least five experiments performed in triplicate for each standard. Representative standard curves are shown in Figure 1. Comparison of the sensitivity of the assays using EP1536Y and the anti-human α-syn (MSD) antibodies (Table 1) showed that when the former was used as capture and the latter for detection, the sensitivity was substantially higher (LLoD = 6 pg/mL, LLoQ = 14 pg/mL) than the reciprocal configuration (LLoD = 15 pg/mL, LLoQ = 75 pg/mL). The two configurations had comparable signal-to-baseline (*S*/*B*) and signal-to-noise (*S*/*N*) ratios (Table S1). In comparison, when mAb MJFR1 was used for capture and EP1536Y for detection, the sensitivity was even lower (LLoD = 32 pg/mL, LLoQ = 97 pg/mL (Table 1)), and the S/B and S/N values also were substantially lower than those of the two first antibody configurations (Table S1). Therefore, we did not test the reciprocal configuration. All three configurations showed excellent specificity for pS^129^-α-syn relative to its unphosphorylated form (Figure 1). Of note, the most sensitive configuration, using biotin-EP1536Y for capture and MSD’s anti-human α-syn antibody for detection, also had the best reproducibility compared to the other configurations. The intra- and interassay CV values were 3.9% and 7.2%, respectively, for this configuration, which also had the widest dynamic range. Thus, this configuration was chosen for subsequent experiments, in which we sought to demonstrate the utility of the assay for the analysis of biological samples. The calculated concentrations, total error (TE%), and relative error (RE%) for the three antibody configurations in Table 1 are given in Tables S2–S4, respectively. The use of a higher biotin-EP1536Y capture antibody concentration of 5 μg/mL compared to 2 μg/μL did not affect the ECL signal intensity (Table S5).

### Determination of pS^129^-α-syn Levels in Biological Samples

**Figure 2:**
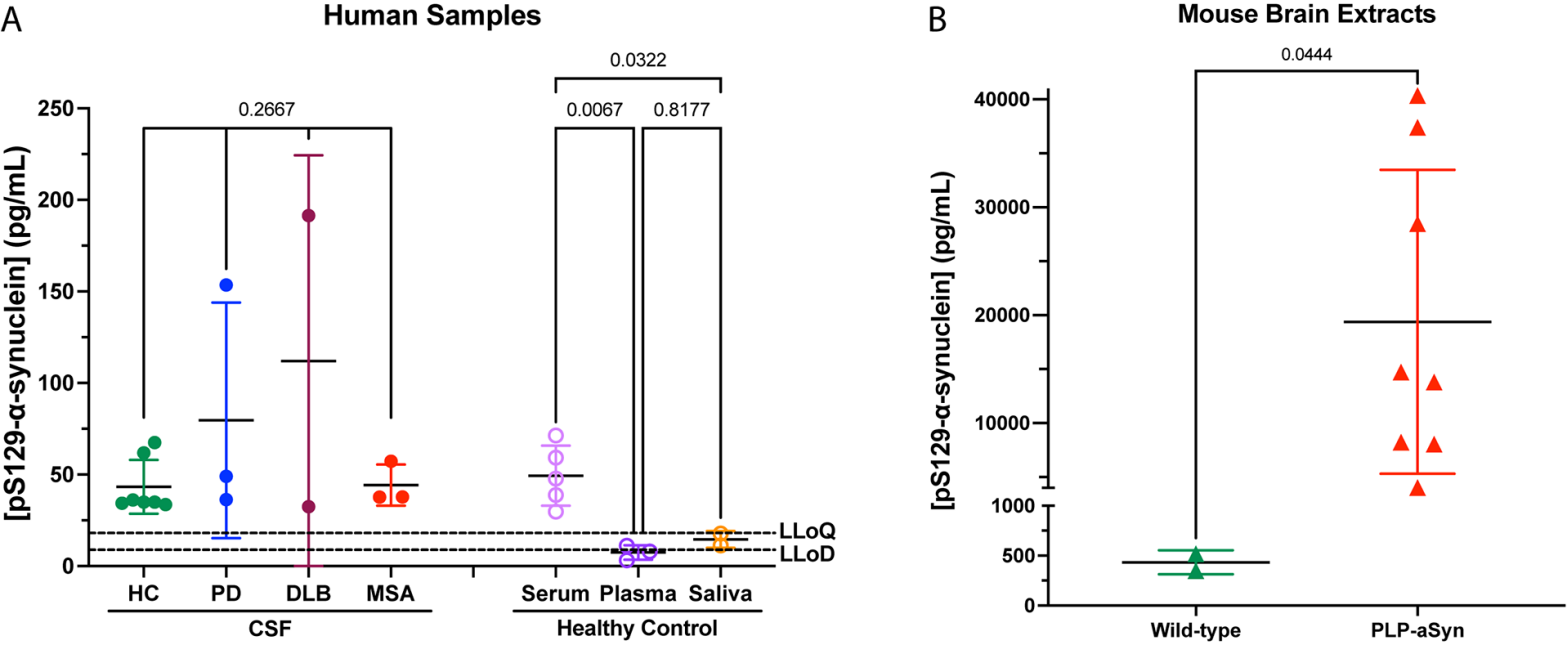
Measurement of pS^129^-α-syn in human and mouse samples. All measurements were performed using biotinylated EP1536Y for capture and MSD’s Sulfo-tagged anti-human-α-syn antibody for detection. (A) Measurement of pS^129^-α-syn in human CSF, serum, plasma, and saliva. HC, healthy control; PD, Parkinson’s disease; DLB, dementia with Lewy bodies; MSA, multiple system atrophy; LLoD, lower limit of detection; LLoQ, lower limit of quantitation. The *p*-values were calculated by a one-way ANOVA with post hoc Tukey test separately for the CSF samples and the serum, plasma, and saliva samples. (B) Comparison of pS^129^-α-syn concentrations in brain extracts from wild-type and MSA model mice. The *p*-values were calculated by Mann–Whitney test. The data are shown as the mean ± SD.

We determined the concentration of pS^129^-α-syn in CSF samples from seven healthy control subjects as well as patients with synucleinopathies (3 PD, 2 DLB, 3 MSA). Due to an apparent moderate matrix effect seen in the CSF (see below), the measured values were corrected using the method of standard addition.^23^ The pS^129^-α-syn concentration levels were variable (Figure 2A), and the small sample numbers did not allow statistically meaningful comparison among the groups. In different samples of commercial, pooled, human serum we determined pS^129^-α-syn levels to be 58 ± 23 pg/mL, whereas in human plasma the concentration was 7 ± 3 pg/mL (*p* = 0.0067 compared to serum, one-way ANOVA, Figure 2A), suggesting that the anticoagulants in the plasma might interfere with the assay. Measurement of pS^129^-α-syn concentrations in the saliva of two healthy control subjects yielded an average concentration of 15 ± 3 pg/mL (*p* = 0.0322 compared to serum, one-way ANOVA, Figure 2A). The concentrations measured in plasma and saliva samples were mostly below the LLoQ, and some were below the LLoD. The saliva results likely reflect the fact that samples from healthy people are expected to contain very low levels of pS129-α-syn, whereas those in the plasma suggest a matrix effect as discussed below. We also analyzed brain lysates from wild-type mice and PLP-α-syn mice, a transgenic model of MSA in which α-syn is overexpressed under the PLP promoter,^24^ leading to its accumulation in oligodendrocytes.^25^ The concentration of pS^129^-α-syn in the soluble fraction of mouse brain extracts (5 μg of total protein) from the PLP-α-syn mice was 19 381 ± 13 173 pg/mL, >50-fold higher than in brain extracts from wild-type mice, 433 ± 120 pg/mL (*p* = 0.0444, Mann–Whitney test, Figure 2B). These findings are in agreement with the known accumulation of the phosphorylated protein in the brains of the MSA-model mice,^26^ demonstrating the utility of the assay for mouse experiments. Finally, we isolated neuronal EVs (nEVs) and oligodendroglial EVs (oEVs) from the serum of healthy controls, patients with PD, and patients with MSA, as described previously,^27^ and measured the pS^129^-α-syn levels in these samples. The analysis showed concentrations between 1.5 and 248.9 pg/mL in nEVs and between 2.2 and 379.9 pg/mL in oEVs. When the measurements of pS^129^-α-syn in the CNS-originating EVs were combined with the previous analysis of total α-syn,^27^ the separation improved among all the groups,^28^ demonstrating the importance and utility of the new assay.

### Dilution Linearity and Spike Recovery

**Figure 3:**
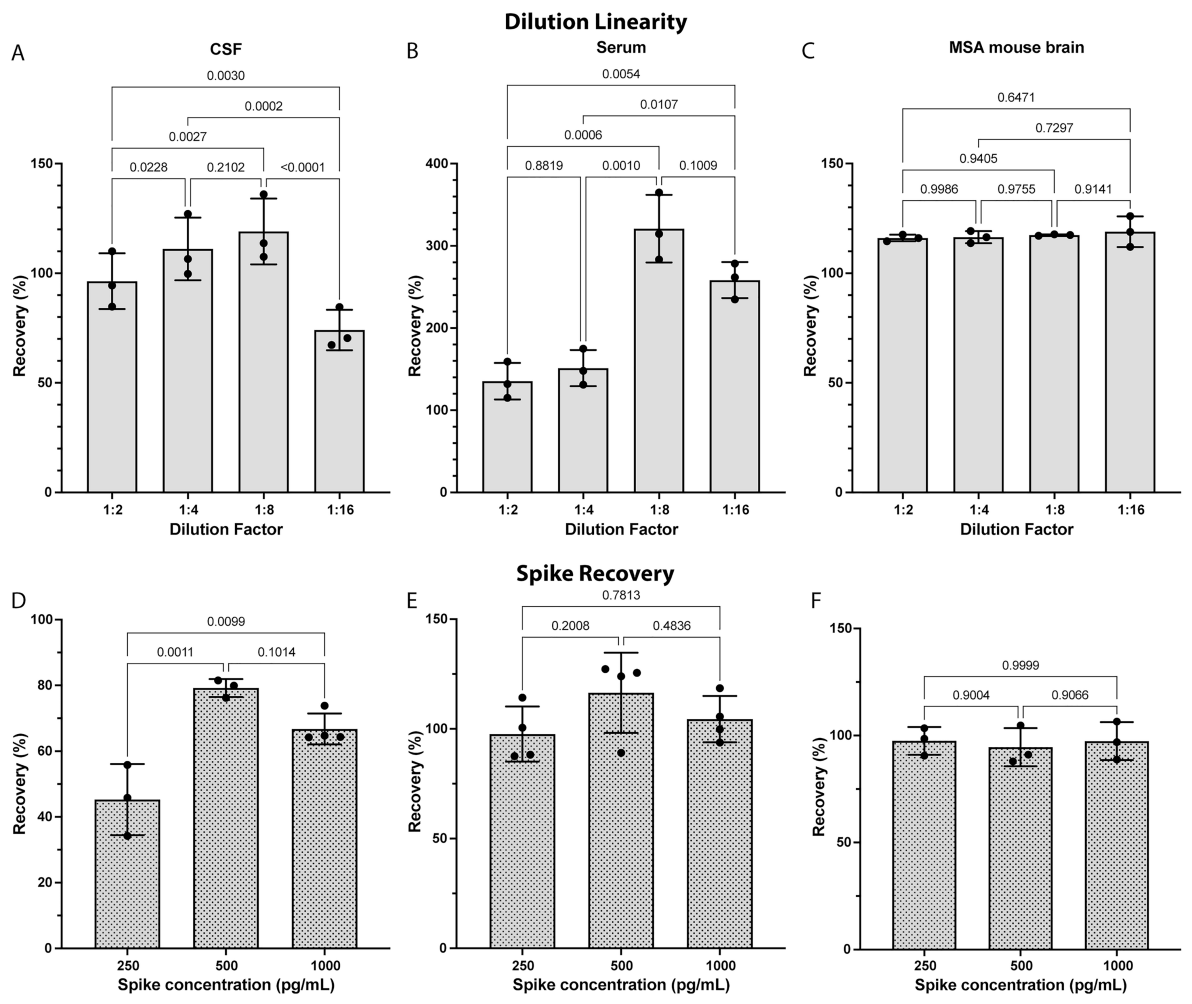
Dilution linearity and spike recovery. (A–C) Dilution linearity within a 2-fold dilution series in (A) human CSF from a patient with DLB, (B) pooled human serum, and (C) extracts of PLP-α-syn mouse brain. The experiments were analyzed by repeated-measure one-way ANOVA with Tukey’s multiple comparisons test with a single pooled variance. (D–F) Spike recovery rates after the addition of 250, 500, or 1000 pg/mL semisynthetic pS^129^-α-syn to (D) human CSF from a patient with DLB, (E) pooled human serum, and (F) extracts of PLP-α-syn mouse brain. The experiments were analyzed by ordinary one-way ANOVA with Tukey’s multiple comparisons test with a single pooled variance. The data are shown as the mean ± SD.

Next, we tested the dilution linearity and spike recovery using biological samples, different from those used in the previous experiments, including human CSF from a patient with DLB (undiluted concentration 113 ± 14 pg/mL), pooled human serum (undiluted 39 ± 9 pg/mL), and a PLP-α-syn mouse brain lysate (undiluted 14 265 ± 457 pg/mL). Acceptable recovery rates were defined as 100 ± 20%. The experiments showed reasonable linearity in the CSF up to 1:8 (Figure 3A) and in the serum up to 1:4 dilution (Figure 3B), likely reflecting the drop of the concentration at higher dilutions below the LLoQ. The dilution linearity was consistent for the PLP-α-syn mouse brain lysate samples (Figure 3C) because of the high concentration of pS^129^-α-syn in these samples, maintaining the concentration well above the LLoQ at all dilutions.

Spike recovery was tested in the same CSF, serum, and MSA mouse-brain extract samples at three concentrations of 250, 500, and 1000 pg/mL. These concentrations were chosen to cover the lower end of the dynamic range, in view of the low concentrations we detected in the human samples (Figure 2). The recovery in CSF samples was low, particularly in the samples spiked with low concentrations (Figure 3D), suggesting that CSF components partially interfere with the signal and therefore the concentrations measured in this medium might be an underrepresentation of the actual pS^129^-α-syn concentration. To address this issue, as mentioned above, we used the method of standard addition to correct the pS^129^-α-syn concentrations measured in the CSF. Good recovery was observed in both the serum (Figure 3E) and mouse-brain extract (Figure 3F) samples.

### Evaluation of pS^129^-α-syn Standard Stability

During the course of the work described above, we observed on multiple occasions deterioration of the signal of the pS^129^-α-syn standard within 1–2 weeks of storage, prompting a detailed investigation to identify the possible causes of this issue and potential solutions. We compared reconstitution of the protein in ddH_2_O or TBS, pH 7.4, with or without pretreatment with trifluoroacetic acid (TFA), filtered through 10 or 100 kDa MWCO filters to remove preformed aggregates, and used 1.0 mg/mL or lower concentrations of the standard protein for the stock solution. In all cases, the solution was aliquoted into single-use aliquots and stored at −80 °C until it was used in the assay. When this solution was used immediately after preparation, the ECLIA signal for the highest-concentration standard, 100 ng/mL, was between 4 × 10^5^ and 1.1 × 10^6^. This level of variability is similar to that reported by MSD for their total α-syn ECLIA kits. However, on multiple occasions, within 1–2 weeks of preparing the stock solution, the signal for this standard deteriorated by 10- to 20-fold, raising concern that the dynamic range of the assay would be too low for meaningful measurement and comparison among experiments would become difficult.

**Figure 4:**
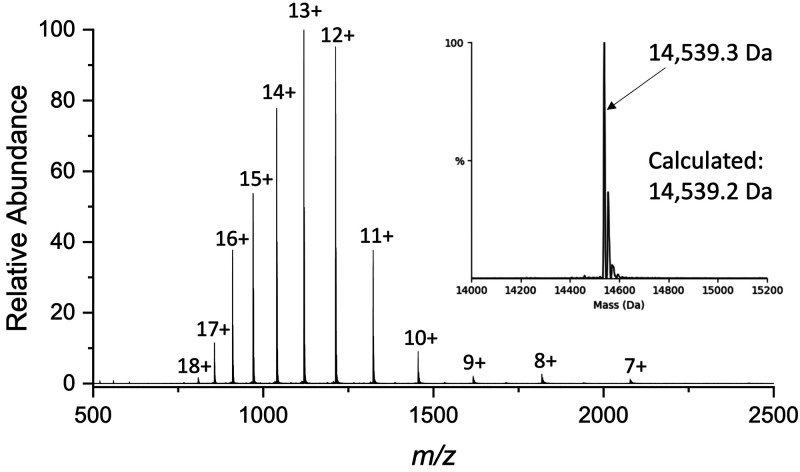
Mass spectrometry analysis of the pS^129^-α-syn standard: an ESI mass-spectrum of pS^129^-α-syn. The inset shows the deconvoluted spectrum corresponding to a single protein species with the correct mass of pS^129^-α-syn.

**Figure 5:**
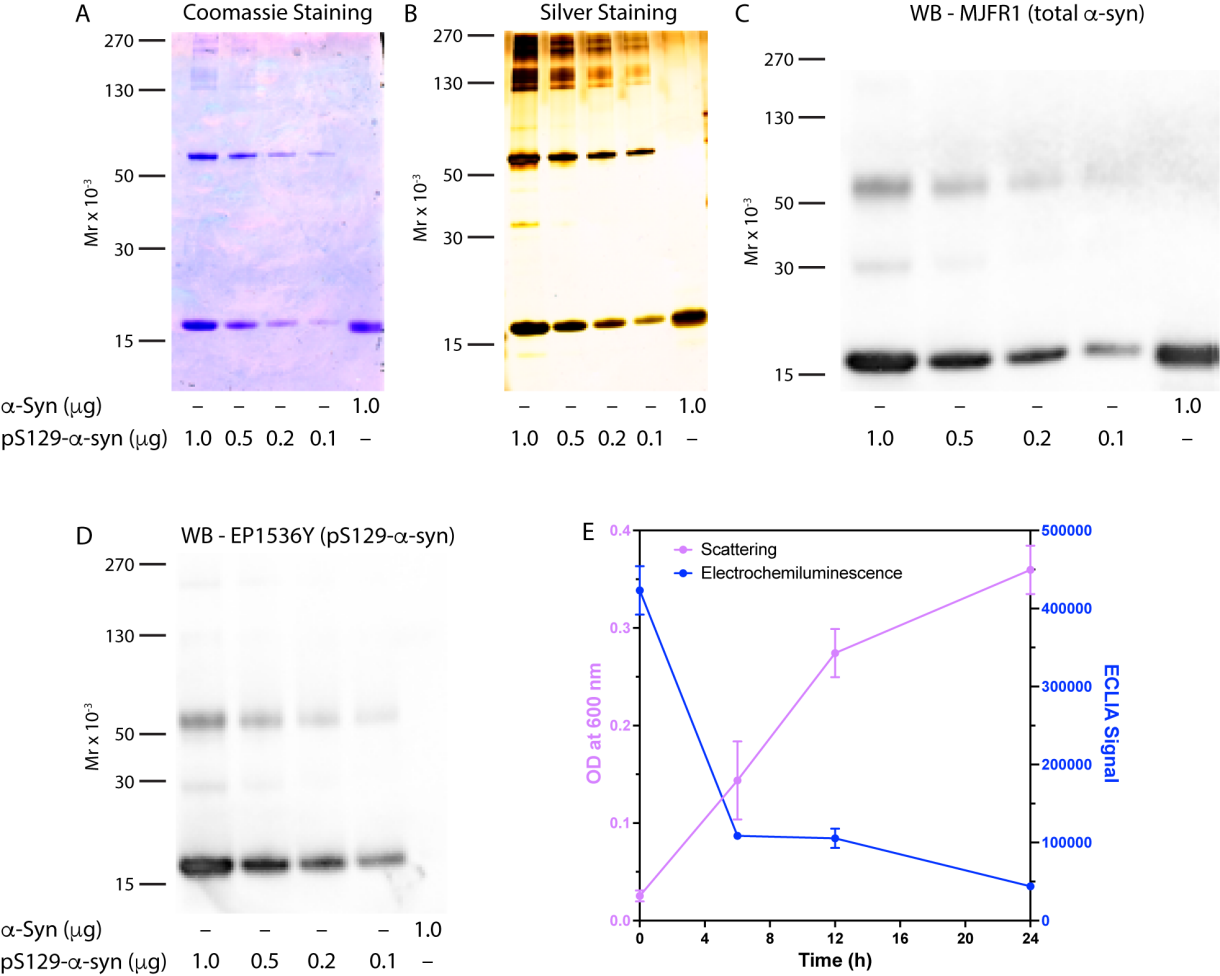
The pS^129^-α-syn standard aggregates when prepared using nonoptimized conditions. (A–D) Different quantities of the semisynthetic pS^129^-α-syn standard were fractionated, and unphosphorylated α-syn was used as a control. (A) Coomassie Blue staining shows the presence of oligomers, presumably tetramers, and larger aggregates of pS^129^-α-syn but not of unphosphorylated α-syn. (B) Higher-sensitivity visualization by silver staining. (C) Western blot analysis probed with the anti-α-syn antibody MJFR1. (D) Western blot analysis probed with the anti-pS^129^-α-syn antibody EP1536Y. The gel migration of molecular weight markers is shown on the left in each panel. (E) 100 ng/mL pS^129^-α-syn was incubated at 37 °C for the indicated times, at which turbidity was measured as absorbance (scattering) at 600 nM and the electrochemiluminescence signal was measured as described in the Experimental Section.

In view of the observed signal deterioration during the storage of the diluted and aliquoted protein standard, we tested whether it might have lost the phosphate group on Ser^129^. Examination of the protein by ESI-MS revealed that it had the intact mass with the expected phosphorylation (Figure 4), ruling out this option. Thus, we hypothesized next that the loss of signal could be due to oligomerization or aggregation of the protein during the preparation process, creating seeds that would promote further rapid aggregation, even when the protein is stored at −80 °C. Indeed, under most of the conditions we used, we observed high-molecular-weight bands of pS^129^-α-syn using SDS–PAGE fractionation followed by Coomassie Blue staining (Figure 5A), silver-staining (Figure 5B), or Western blots probed with antibodies MJFR1 (Figure 5C) or EP1536Y (Figure 5D). Unphosphorylated α-syn was used as a control in these experiments and migrated as a monomer only (Figure 5A–C). Changing the solution from ddH_2_O to TBS and filtering the stock solution did not resolve the issue. Reducing the concentration of the stock solution to 0.29 mg/mL, as recommended by Cariulo et al.,^12^ reduced the aggregation, but the signal loss was still observed if the TFA used for the initial dissolution of the protein powder was not removed completely.

EP1536Y has been used in immunohistochemistry studies to detect aggregated pS^129^-α-syn in LBs or GCIs.^19^ Therefore, the interpretation of the signal loss in the ELICA assay as reflecting aggregation was counterintuitive. To further examine this idea, we incubated the protein with agitation for up to 24 h, monitored occasionally its aggregation using a turbidity assay, and measured the signal of the highest standard (100 ng/mL) at the same time points (Figure 5E). These experiments revealed that the protein indeed aggregated concomitant with a substantial decrease in the ECLIA signal.

**Figure 6:**
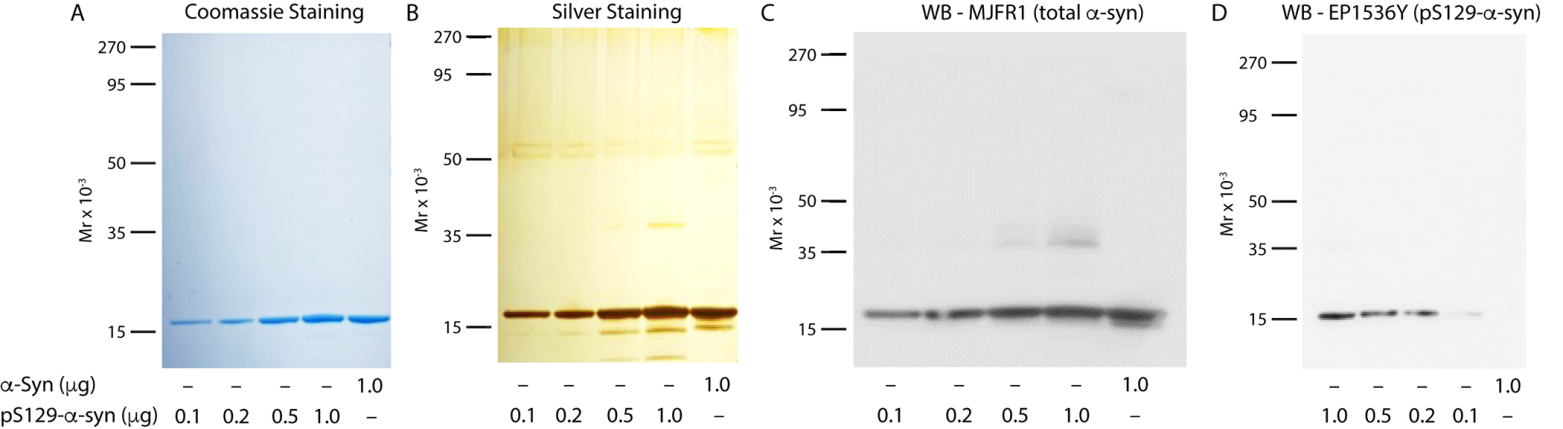
SDS–PAGE and Western-blot analysis of the pS^129^-α-syn standard prepared using optimized conditions. Different quantities of the semisynthetic pS^129^-α-syn standard were fractionated, and unphosphorylated α-syn was used as a control. (A) Coomassie Blue staining shows an absence of oligomers of pS^129^-α-syn. (B) Higher-sensitivity visualization of the same gel by silver staining shows minor bands of a putative dimer in the 0.5 and 1.0 μg pS^129^-α-syn lanes. Minor degradation products are also observed under the monomer band. Bands between 50 and 60 kDa likely are keratin contamination and are not related to the analyzed proteins. (C) Western blot analysis probed with the anti-α-syn antibody MJFR1 showing minor putative dimer bands in the 0.5 and 1.0 μg pS^129^-α-syn lanes. (D) Western blot analysis probed with the anti-pS^129^-α-syn antibody EP1536Y. The gel migration of molecular weight markers is shown on the left in each panel.

Following the protocol published by Cariulo et al.^12^ precisely and ensuring that the TFA was removed completely was necessary for preventing seed formation and allowing the protein to remain unaggregated for prolonged storage. Under these conditions, weekly repeated testing of the standard curve and positive control samples (MSA mouse-brain extract) yielded consistent data over one month without apparent signal loss. Analysis of the protein prepared in this manner using SDS–PAGE followed by Coomassie Blue staining (Figure 6A), silver-staining (Figure 6B), or Western blots probed with MJFR1 (Figure 6C) or EP1536Y (Figure 6D) showed an absence of the previously observed high-molecular-weight bands.

## Discussion

High throughput measurement of disease-relevant, post-translationally modified forms of α-syn with high sensitivity currently is an unmet need for clinical and biomarker studies. To address this need, we developed a novel assay based on the MSD ECLIA platform that detects and quantifies pS^129^-α-syn at low pg/mL concentrations in various biological samples. The assay does not detect unphosphorylated forms of α-syn up to concentrations of >10 ng/mL (Figure 1). The specificity and high sensitivity of the assay are achieved when using monoclonal antibody EP1536Y for the capture of the analyte followed by detection by the Sulfo-Tag anti-α-syn antibody provided in MSD’s total α-syn kit. Compared to the two other antibody combinations/configurations we tested, this capture and detection combination provided not only the highest sensitivity but also the highest dynamic linear range and best reproducibility. In agreement with this finding, other groups showed that the EP1536Y antibody is highly specific for pS^129^-α-syn and could detect it robustly even in the presence of other post-translational modifications in close proximity to Ser^129^ compared to other evaluated anti-pS^129^-α-syn antibodies.^15,18−20^ However, interference of other post-translational modifications and phosphorylation at other sites of α-syn were not assessed in our assay. Lashuel and colleagues reported that EP1536Y does detect α-syn phosphorylated at both Tyr^125^ and Ser^129^ and does not detect pS^129^-α-syn truncated after residue 133 or 135. In addition, the antibody showed a reduced signal when tested for binding of α-syn fibrils phosphorylated at Ser^129^ and nitrated at Tyr^125^, Tyr^133^, and Tyr^136^ compared to fibrils of pS^129^-α-syn itself.^18^ The contribution of these other post-translationally modified α-syn forms to the signal in the biological samples we tested here or to the levels of pS^129^-α-syn measured in previous studies currently is not known.

When comparing the ECLIA platform described here with other published methods for quantifying pS^129^-α-syn in biological fluids, it is important to note the lack of consensus in the data from different groups. For example, Wang et al. reported pS^129^-α-syn concentrations of 58–80 pg/mL in the CSF of patients with PD or MSA using a bead-based Luminex assay,^10^ whereas Majbour et al. found substantially higher pS^129^-α-syn concentration levels, 181–275 pg/mL in the CSF of healthy individuals and 207–296 pg/mL in the CSF of patients with PD determined by a sandwich ELISA.^7^ These assays used casein kinase II to phosphorylate recombinant α-syn, which was used as a standard, and Wang et al. diluted their samples by 3/4, whereas Majbour et al. did not. The corrected pS^129^-α-syn concentrations we detected in CSF samples were between 32 and 191 pg/mL (Figure 2), in rough agreement with both studies. In contrast, a study by Cariulo et al. did not detect pS^129^-α-syn in pooled commercial CSF despite using a highly sensitive Singulex Erenna immunoassay with a LLoD of 0.15 pg/mL.^12^ This apparent discrepancy may be explained in part by the differences between the particle-based digital single-molecule counting in the Singulex Erenna and the electrochemiluminescence signal measured by ECLIA, which allowed us to use a different sample preparation process that did not require transfer of beads multiple times to different tubes/plates, followed by the appropriate wash steps, which might have helped reduce the loss of signal by nonspecific adsorption to surfaces. In addition, the antibody we used, EP1536Y, has been reported to have higher sensitivity and specificity compared to the one used by Cariulo et al. MJF-R13 (8-8),^19^ presumably increasing further our ability to detect pS129-α-syn in the CSF. In contrast, using IP-MS/MS, Lashuel and co-workers also did not detect pS^129^-α-syn in the CSF of patients with PD and healthy controls, highlighting the difficulty and inconsistency in measuring this α-syn species in CSF.^30^

To our knowledge, one previous study tested pS^129^-α-syn in serum using a modified paired-surface plasma-wave biosensor and reported concentrations in the range 500–5000 pg/mL in HC and 4000–12000 pg/mL in patients with PD.^31^ The large differences between the technique used in that paper and the ECLIA used here make comparison with our data difficult. Our data suggest that plasma components may interfere with the assay (Figure 2). The plasma signal in our assay was an order of magnitude lower than the signal observed in serum samples. The two fluids differ mainly by the presence of anticoagulants in plasma and possibly coagulation factors, e.g., fibrinogen, which may interfere with the assay. As both types of samples were commercial, pooled biofluids and not taken from the same persons, a direct comparison was not possible. Previous studies by Foulds et al. in two separate cohorts found 200–600 ng/mL ^6^ and 143 ± 532 ng/mL pS^129^-α-syn ^14^ in control human plasma, several orders of magnitude above the concentrations we measured. However, these results also were over 3 orders of magnitude higher than the concentrations we found in serum, suggesting that assay differences likely were the main reason for these large discrepancies. In the study by Cariulo et al., pS^129^-α-syn levels in the range 480–1223 pg/mL were found for clinically obtained and commercially pooled plasma samples.^12^ The large discrepancy in values may be attributed to the differences in the assays themselves, batch variation, sample source, sample processing, and the antibody pairs used, as discussed above.

The MSD ECLIA platform used for this assay is a highly sensitive, versatile, and widespread technology, and similar to the other platforms, allows duplexing of assays, for example, for quantification of pS^129^-α-syn and unphosphorylated α-syn together. Creating such a duplex assay is outside the scope of the current work, but we expect to develop it in the future. Notwithstanding the limitations discussed above, our results suggest that the pS^129^-α-syn ECLIA is an attractive tool for measuring pS^129^-α-syn in CSF, serum, saliva, potentially other biofluids, brain lysates, or other experimental systems, such as cell cultures and animal models. Nevertheless, cross-validation of our assay by other groups, thorough validation of the used antibodies, and strictly standardized sample preparation protocols are crucial for achieving reliable and reproducible results. We anticipate that future commercial kits will provide small aliquots of the standard that can be prepared freshly for single use. Until such assays become available, researchers interested in using the assay are advised to follow carefully the protocol published by Cariulo et al.^12^ to ensure that the semisynthetic pS^129^-α-syn, which currently is sold by Proteos only in 1 mg portions, is not lost due to aggregation shortly after its preparation.

## Experimental Section

### Materials

Semisynthetic pS^129^-α-syn was obtained from Proteos Inc. (Kalamazoo, MI). Anti-pS^129^-α-syn monoclonal antibody EP1536Y (ab209422) and anti-α-synuclein monoclonal antibody MJFR1 (ab138501) were procured from Abcam (Cambridge, UK). Small-spot streptavidin-coated 96-wells ELISA plates (L45SA), assay diluent (R50AM), recombinant human α-syn calibrator (C01WK), biotinylated anti-total α-syn antibody, SULFO-TAG anti-human α-syn antibody, GOLD SULFO-TAG NHS-ester conjugation pack (R31AA), and read buffer (R92TC) were obtained from Meso Scale Discovery (Rockville, MD). Phosphate-buffered saline (PBS), Tris-buffered saline (TBS), and biotin quantitation kit were from Thermo Fisher Scientific (Waltham, MA).

### Biological Samples

Pooled human serum and pooled human plasma were procured from Innovative Research (Novi, MI). Cerebrospinal fluid samples were collected post-mortem from deceased patients with synucleinopathies or controls without a neurological disease whose diagnosis was determined by a neuropathological examination. The samples were obtained from the UCLA Division of Neuropathology (2 PD, 2 DLB, 3 MSA), UC Irvine Institute for Memory Impairments and Neurological Disorders (MIND, 7 healthy controls), and Banner Sun Health Research Institute (BHSRI), Sun City, AZ (1 PD). Protocols for CSF collection post-mortem were similar in the three institutions with small variations. At UCLA and BHSRI, CSF was drawn into 30 mL polypropylene syringes using 8 cm long 18-gauge needles. UCI MIND used similar needles but 20 mL syringes. At UCLA and BHSRI the CSF was transferred to 15 mL Falcon tubes and centrifuged at low speed to get rid of particulate matter. Then, the supernate was aliquoted into 0.5 mL aliquots. AT UCI MIND, the collected CSF was placed on ice until processing and then aliquoted into 0.25 mL aliquots. The aliquots in all three institutions were stored at −80 °C until use. Mouse brain extracts were from PLP-α-syn mice, a model of experimental MSA, or wild-type littermates and were obtained as described previously.^24^ All the biological samples were treated with 1× Halt protease and phosphatase inhibitor cocktail (Thermo Fisher Scientific).

### Preparation of Standard

The pS^129^-α-syn standard was prepared according to the protocol published previously by Cariulo et al.^12^ Briefly, the lyophilized protein was weighed and dissolved in 100% trifluoroacetic acid (TFA) purchased from Alfa Aesar (Haverhill, MA). 1 μL of TFA was added per 25 μg of protein. The TFA was evaporated completely in a fume hood for 1 h, and the protein then was dissolved in TBS (50 mM Tris, 150 mM NaCl, pH 7.4) at a concentration of 20 μM. The solution was filtered through a 100 kDa cutoff filter from Pall (Show Low, AZ), and protein concentration was measured using a bicinchoninic acid (BCA) assay (Thermo Fisher Scientific). 1% (v/v) Tween-20 (Fisher BioReagents, Pittsburgh, PA) was added, and the solution was aliquoted and stored at −80 °C.

### Biotinylation and Sulfonation of Antibodies

For biotinylation of antibody EP1536Y, 40 μL of a 0.90 mg/mL solution of the antibody was incubated with 1.61 μL of 11 mM Sulfo-NHS-biotin (50-fold molar excess) on ice for 2 h according to the manufacturer’s protocol (EZ-Link Micro Sulfo-NHS-LC-biotinylation kit, Thermo Fisher Scientific). Excess free biotin was removed using a 0.5 mL of 7 kDa MWCO Zeba spin desalting column (Thermo Fisher Scientific). 200 μL of ultrapure water (purified using a Milli-Q system, Millipore, Burlington, MA) was added as a stacker volume for elution of the biotinylated EP1536Y antibody. 40 μL of 1.167-mg/mL solution of antibody MJFR1 was biotinylated using the same biotinylation kit. Biotinylation levels were evaluated using a fluorescence biotin quantitation kit (Thermo Fisher Scientific). For sulfonation, 40 μL of a 0.90 mg/mL of EP1536Y solution was incubated with Sulfo-TAG NHS-ester (MSD) according to the manufacturer’s instructions. The protein concentration of all antibody conjugates was determined using a Pierce BCA assay kit (Thermo Fisher Scientific). The antibody solutions were aliquoted and stored at 4 °C until use.

### Preparation of Biological Samples

CNS-originating extracellular vesicles (EVs) were isolated from the serum of HC, patients with PD, and patients with MSA as described previously.^27^ For analysis of pS^129^-α-syn in the CSF of deceased patients with different synucleinopathies and healthy controls, as well as in CNS-originating EVs isolated from human serum, 15 μg of total protein, determined using a BCA assay, in 25 μL of Diluent 49 (MSD) was analyzed. Saliva samples obtained from healthy volunteers were centrifuged briefly at 10 000*g* for 10 min at 4 °C, the clear supernates were collected, and a final volume of 25 μL was used for analysis. 25 μL of clear pooled human serum or plasma containing 1× Halt protease and phosphatase inhibitor cocktail was loaded directly onto the wells. MSA or wild-type mouse brain lysates (5 μg total protein) were diluted in 25 μL of Diluent 49 for ECLIA analysis.

### ECLIA

The assay was developed using MSD gold 96-well small-spot streptavidin SECTOR ELISA plates. Capture and detection antibody concentrations were determined based on the information available on MSD’s website for validation of the human α-syn assay. We found that a concentration of 2 μg/mL antibody was sufficient for the capture antibody to saturate the wells of the streptavidin-coated plates (Table S5). Biotinylated-antibody stock solutions were diluted in 1% (w/v) biotin-free bovine serum albumin (bf-BSA) in TBS containing 0.1% (v/v) Tween-20 (TBS-T) to reach a concentration of 2 μg/mL EP1536Y, MJRF1, or MSD’s 1× anti-α-syn capture antibody. Dilutions of the semisynthetic pS^129^-α-syn, recombinant human α-syn standards, and biological samples were made in Diluent 49 (MSD), which also was used as a “zero” calibrator (blank). 150 μL of 3% (w/v) bf-BSA in TBS-T was added to each well, and the plates were incubated with shaking at 800 rpm at RT for 1 h. The blocking solution then was removed, and 25 μL of 2 μg/mL or 1× biotinylated capture antibodies were added to each well. The plates were incubated with shaking at 800 rpm at RT for 1 h and then washed three times with 150 μL of TBS-T per well. Subsequently, 25 μL of SULFO-TAG detection antibody and 25 μL of samples or standards were added to the wells and the plates were incubated further with shaking at 800 rpm at RT for 2 h. After washing three times with 150 μL of TBS-T per well, 150 μL of 1× read buffer (MSD) was added to each well and the plates were read immediately using a Sector S600 reader (MSD). The data were analyzed using Discovery Workbench 4.0 software (MSD) and quantified with reference to freshly prepared standard curves.

### Spike Recovery

To test spike recovery, a human CSF (DLB) sample, pooled serum, a PLP-α-syn mouse brain lysate, or Diluent 49 as a control were spiked with 250 pg/mL (low spike), 500 pg/mL (medium spike), or 1000 pg/mL (high spike) of the semisynthetic pS^129^-α-syn standard. 25 μL of each spiked or unspiked sample was analyzed. The spike recovery rate was calculated as the ratio of the measured and the calculated concentration.

### Dilution Linearity

To test the dilution linearity, biological samples were serially diluted 2-fold four times in Diluent 49 (MSD). The final volume of each sample was 25 μL. Final concentrations were measured and compared to the calculated concentrations based on the appropriate dilution factor.

### Electrospray Ionization Mass Spectrometry (ESI-MS)

The standard protein was dissolved at 1 mg/mL in ddH_2_O, buffer-exchanged into 20 mM ammonium acetate, pH 6.8, using 10 kDa MWCO Amicon centrifugal filters (Millipore Sigma, Burlington, MA), and diluted to 10 μM in the same buffer. The solution was electrosprayed using pulled nanoESI needles onto a Bruker 15T Solarix Fourier-transform ion cyclotron mass spectrometry system. The capillary voltage was set to 800 V and the temperature to 180 °C. The deflector plate was set to 160 V, the capillary exit to 100 V, the funnel voltage to 90 V, and the skimmer to 50 V. One-hundred scans were collected to obtain the spectrum. The deconvolved spectrum was created using UniDec.^32^

### SDS–PAGE and Staining

1, 0.5, 0.25, or 0.125 μg of pS^129^-α-syn and 1 μg of unphosphorylated α-syn were fractionated using Sure PAGE 4–20% gradient Bis-Tris gels (GenScript). Samples were prepared by mixing the protein solution with sample buffer (GenScript) and heated at 95 °C for 10 min. Gels were stained in 0.1% (w/v) Coomassie Brilliant Blue (Thermo Fisher Scientific) in 40% (v/v) methanol and 10% (v/v) acetic acid for 1 h at RT and then destained in the same solution excluding Coomassie Brilliant Blue. Silver-staining was performed using the SilverXpress silver staining kit (Invitrogen) following the manufacturer’s protocol.

### Immunoblotting

Following SDS–PAGE fractionation, the proteins were transferred onto polyvinylidene fluoride (PVDF) membranes (Thermo Fisher Scientific) for 1 h at 25 V on ice using XCell II blot modules (Invitrogen). The membranes were blocked using 5% (w/v) nonfat dry milk in TBS-T (blocking solution) for 1 h at RT and then incubated with either MJFR1 or EP1536Y at a 1:1000 dilution in blocking solution overnight at 4 °C with gentle agitation. The membranes then were washed in TBS-T thrice and incubated with HRP-conjugated goat anti-rabbit antibody (Thermo Fisher Scientific) at 1:10000 dilution in blocking solution for 1 h at RT, developed using SuperSignal West Pico PLUS chemiluminescent substrate (Life Technologies), and visualized using an Azure Biosystems c300 gel imager.

### Turbidity Assay

20 μM pS^129^-α-syn was incubated for 0, 6, 12, or 24 h at 37 °C with agitation at 300 rpm. 2 μL of the protein was diluted in 48 μL of TBS, and absorbance at 600 nm was recorded as a measure of turbidity (Ultrospec 2000, Pharmacia Biotech).

### Data Processing and Statistical Analysis

The sensitivity and dynamic range of each antibody combination were evaluated by determination of the limit of blank (LoB), lower limit of detection (LLoD), lower limit of quantification (LLoQ), and upper limit of quantification (ULoQ). The applied definitions for the LoB, LLoD, LLoQ, and ULoQ are described in Table 2. Data were processed using MSD Discovery Workbench 4.0 software and Prism 9.4 (GraphPad, USA). Standard curves and sample concentrations were calculated using a four-parameter fit and plotted as the mean ± standard deviation of four replicates. The intra-assay coefficient of variation (CV) was calculated between standard concentrations measured on the same plate, whereas interassay CV was calculated in three independent experiments using different plates. Signal/background (*S*/*B*) and signal/noise (*S*/*N*) ratios were calculated as averages of three standard concentration points within the linear range of the assay for each antibody pair. Total percent error (TE%) was calculated as ((calculated concentration – actual concentration) + 2 SD)/actual concentration) × 100. Relative percent error (RE%) was calculated as ((calculated concentration – actual concentration)/actual concentration) × 100 (see Supporting Information). Standard error for the method of standard addition was calculated according to Bruce and Gill^33^ using the formula
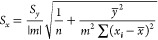


**Table 2:** Definitions of Assay Parameters^34^

LoB	calculated concentration based on the mean zero calibrator signal + 1.645 × the standard deviation (SD) of the zero calibrator
LLoD	calculated concentration based on the signal + 1.645 × SD (lowest calibrator) above the LoB
LLoQ	calculated concentration based on the signal + 10 × SD (blank) above the zero calibrator
ULoQ	calibrator concentration at the upper limit of the linear range on a logarithmic scale

## Supporting Information Available

Tables providing signal/background (*S*/*B*) and signal/noise (*S*/*N*) ratios, calculated concentrations, total error, and relative error for pS^129^-α-syn and α-syn in assays using different antibody pairs and a comparison of pS^129^-α-syn standard curves using 2 or 5 μg/μL capture antibody (PDF)
