# Supplementary material for: Development of a Novel Electrochemiluminescence ELISA for Quantification of α-Synuclein Phosphorylated at Ser129 in Biological Samples
Source: ACS Chem Neurosci. 2023 Mar 15;14(7):1238–48. doi: 10.1021/acschemneuro.2c00676 (PMC10080651; doi:10.1021/acschemneuro.2c00676)
Supplement: Supplementary file 1 — cn2c00676_si_001.pdf [file cn2c00676_si_001.pdf]

Supplementary information

Development of a novel electrochemiluminescence ELISA for quantification of α-synuclein phosphorylated at Ser^{129} in biological samples

Suman Dutta, PhD[1‡], Simon Hornung[1‡†], Hash Brown Taha[1,2‡], Karl Biggs[1] Ibrar Siddique[1], Lea M. Chamoun[1], Hedieh Shahpasand-Kroner[1], Carter Lantz[3], Marcos Herrera-Vaquero[4], Nadia Stefanova[4], Joseph A. Loo[3,5], and Gal Bitan, PhD[1,5,6*]

[1]Department of Neurology, David Geffen School of Medicine, [2]Department of Integrative Biology & Physiology, [3]Department of Chemistry and Biochemistry, University of California Los Angeles, CA 90095, USA, [4]Division of Neurobiology, Department of Neurology, Medical University of Innsbruck, Austria, [5]Brain Research Institute and [6]Molecular Biology Institute, University of California, Los Angeles, CA 90095, USA

***To whom correspondence should be addressed:**

Gal Bitan

Department of Neurology,
David Geffen School of Medicine at UCLA,
635 Charles E. Young Drive South/Gordon 451
Los Angeles, CA 90095
E-mail: gbitan@mednet.ucla.edu
Phone: 310-206-2082

‡ These authors contributed equally

† Current address: Division of Peptide biochemistry, TUM School of Life Sciences, Technical University of Munich, 85354 Freising, Germany

Key words: Electrochemiluminescence ELISA, α-synuclein, phosphorylation, pS129, biomarker

Table S1: Signal/background (S/B) and signal/noise (S/N) ratios for pS^{129} α-syn and α-syn of the evaluated antibody pairs.[1]

Capture antibody	Detection antibody	S/B (pS^{129} α-syn)	S/N (pS^{129} α-syn)	S/B (α-syn)	S/N (α-syn)
Biotin-EP1536Y (anti-pS^{129}-α-syn)	Anti-human α-syn-Sulfo tag (MSD)	426	43674	1	86
Biotin-anti-human α-syn (MSD)	EP1536Y-Sulfo tag	651	49217	1	65
Biotin-MJFR1 (anti-human α-syn)	EP1536Y-Sulfo tag	37	2791	1	43

The S/B and S/N ratios are averages of three standard concentration points within the linear range of the assay using each antibody pair.

Table S2: Calculated concentrations, total error (TE)[2] **and relative error (RE)**[3] **of pS^{129}-α-syn ECLIA using EP1536Y for capture and anti-human α-syn antibody (MSD) for detection.**

pS^{129}-α-syn (pg/mL)	Assay 1		Assay 2		Assay 3	TE (%)	RE (%)
	1	2	1	2	1		
66176	77102	65766	68279	71620	66202	18.4	5.7
33088	33119	31361	32100	33769	34979	10.9	3.2
16544	14916	15799	14657	16233	15718	13.6	6.5
8272	7836	8250	7690	7918	8151	8.6	3.7
2068	2269	2148	2266	2244	2004	16.7	7.0
517	613	571	604	605	550	23.2	13.8
129	126	124	122	122	141	16.4	5.3
32	18	23	26	21	32	54.8	25.0
8	5.7	4.4	3.8	4.7	5.9	58.6	38.8
2	1.7	4.0	2.1	2.5	0.8	146.3	41.0
0.5	1.5	2.2	2.1	1.8	1.7	375.1	272.0

TE (%) = ((calculated concentration – actual concentration) + 2 SD)/actual concentration) × 100; RE (%) = ((calculated concentration – actual concentration)/actual concentration) × 100.

Table S3: Calculated concentrations, total error (TE)[2] **and relative error (RE)**[3] **of pS^{129} ECLIA using the Biotin-anti-human α-syn (MSD) - EP1536Y-Sulfo tag antibody pair.**

pS^{129} α-syn (pg/mL)	Assay 1		Assay 2		TE (%)	RE (%)
	1	2	1	2		
66176	71338	65440	64368	69755	13.0	4.3
33088	32655	33723	34581	32114	8.4	2.7
16544	16352	16028	17348	16315	8.7	2.6
8272	8161	8136	7653	8174	8.2	2.9
2068	2096	2112	2118	2094	2.8	1.8
517	527	518	533	506	5.8	1.8
129	128	137	139	132	11.1	4.2
32	19	28	32	26	49.1	18.8
8	14	8.1	6.4	3.5	137.8	38.9
2	n.d.	2.9	0.8	3.2	174.2	66.4
0.5	3.9	0.5	2.1	2.5	836.1	352.9

n.d. – not determined

Table S4: Calculated concentrations, total error (TE)[2] and relative error (RE)[3] of pS^{129} ECLIA using the Biotin-MJFR1 - EP1536Y-Sulfo tag antibody pair.

pS^{129} α-syn (pg/mL)	Assay 1		Assay 2		TE (%)	RE (%)
	1	2	1	2		
66176	60951	65118	64829	69720	13.6	4.2
33088	33826	34340	32593	34348	7.2	2.8
16544	16393	18542	15645	17102	18.4	5.4
8272	9349	7820	7639	8187	22.9	6.8
2068	1631	1791	2338	2130	39.5	12.6
517	397	298	470	585	62.6	22.0
129	468	751	108	97	618.6	196.4
32	7.0	n.d.	15	10	86.8	66.7
8	2.0	n.d.	8.2	28	384.4	108.5
2	39	10	n.d.	17	2198.9	999.1
0.5	0.6	n.d.	2.9	2.9	752.1	317.5

n.d. – not determined

Table S5: ECL signal of pS^{129} α-syn standards using 2 or 5 μg/mL Biotin-EP1536Y capture antibody and MSD Sulfo-tag antibody for detection.

pS^{129} α-syn (pg/mL)	2 μg/mL capture antibody	5 μg/mL capture antibody
10000	107291	103256
2500	17083	16278
625	3529	3507
156	957	902
39	332	317
9.8	152	135
2.4	116	95
0	96	91

Financial Disclosure/Conflict of interests: GB, SD, and KB are co-authors and co-inventors of Patent Application No. 63/482,173 entitled "An electrochemiluminescence ELISA for pS129-α-synuclein."

References

1. Zhang, J. H.; Chung, T. D.; Oldenburg, K. R., A Simple Statistical Parameter for Use in Evaluation and Validation of High Throughput Screening Assays. *J. Biomol. Screen.* **1999,** *4* (2), 67-73.
2. Westgard, J. O.; Carey, R. N.; Wold, S., Criteria for judging precision and accuracy in method development and evaluation. *Clin. Chem.* **1974,** *20* (7), 825-33.
3. Kat, C.-J.; Els, P. S., Validation metric based on relative error. *Math. Comput. Model. Dyn. Syst.* **2012,** *18* (5), 487-520.
